# Supplementary material for: Impact of COVID-19 pandemic on breast and cervical cancer screening in Denmark: A register-based study
Source: eLife. 2023 Mar 21;12:e81605. doi: 10.7554/eLife.81605 (PMC10030107; doi:10.7554/eLife.81605)
Supplement: Supplementary file 1. [file elife-81605-supp1.docx]

Nykøbing Falster Hospital 24 January 2023

**IMPACT OF COVID-19 PANDEMIC ON BREAST AND CERVICAL**

**CANCER SCREENING IN DENMARK:**

**A register-based study**

Mette Hartmann Nonboe^1*^, George Napolitano^2^, Jeppe Bennekou Schroll^3^, Ilse Vejborg^4^  Marianne Waldström^5^ and Elsebeth Lynge^1^

**Supplementary File 1**. Overview of dates within “Months”

| **Month** | **2017** | | **2018** | | **2019** | | **2020** | | **2021** | |
| --- | --- | --- | --- | --- | --- | --- | --- | --- | --- | --- |
|  | **Start date** | **End date** | **Start date** | **End date** | **Start date** | **End date** | **Start date** | **End date** | **Start date** | **End date** |
| **01** | 2017-01-02 | 2017-01-29 | 2018-01-01 | 2018-01-28 | 2018-12-31 | 2019-01-27 | 2019-12-30 | 2020-01-26 | 2021-01-04 | 2021-01-31 |
| **02** | 2017-01-30 | 2017-02-26 | 2018-01-29 | 2018-02-25 | 2019-01-28 | 2019-02-24 | 2020-01-27 | 2020-02-23 | 2021-02-01 | 2021-02-28 |
| **03** | 2017-02-27 | 2017-03-26 | 2018-02-26 | 2018-03-25 | 2019-02-25 | 2019-03-24 | 2020-02-24 | 2020-03-22 | 2021-03-01 | 2021-03-28 |
| **04** | 2017-03-27 | 2017-04-23 | 2018-03-26 | 2018-04-22 | 2019-03-25 | 2019-04-21 | 2020-03-23 | 2020-04-19 | 2021-03-29 | 2021-04-25 |
| **05** | 2017-04-24 | 2017-05-21 | 2018-04-23 | 2018-05-20 | 2019-04-22 | 2019-05-19 | 2020-04-20 | 2020-05-17 | 2021-04-26 | 2021-05-23 |
| **06** | 2017-05-22 | 2017-06-18 | 2018-05-21 | 2018-06-17 | 2019-05-20 | 2019-06-16 | 2020-05-18 | 2020-06-14 | 2021-05-24 | 2021-06-20 |
| **07** | 2017-06-19 | 2017-07-16 | 2018-06-18 | 2018-07-15 | 2019-06-17 | 2019-07-14 | 2020-06-15 | 2020-07-12 | 2021-06-21 | 2021-07-18 |
| **08** | 2017-07-17 | 2017-08-13 | 2018-07-16 | 2018-08-12 | 2019-07-15 | 2019-08-11 | 2020-07-13 | 2020-08-09 | 2021-07-19 | 2021-08-15 |
| **09** | 2017-08-14 | 2017-09-10 | 2018-08-13 | 2018-09-09 | 2019-08-12 | 2019-09-08 | 2020-08-10 | 2020-09-06 | 2021-08-16 | 2021-09-12 |
| **10** | 2017-09-11 | 2017-10-08 | 2018-09-10 | 2018-10-07 | 2019-09-09 | 2019-10-06 | 2020-09-07 | 2020-10-04 | 2021-09-13 | 2021-10-10 |
| **11** | 2017-10-09 | 2017-11-05 | 2018-10-08 | 2018-11-04 | 2019-10-07 | 2019-11-03 | 2020-10-05 | 2020-11-01 | 2021-10-11 | 2021-11-07 |
| **12** | 2017-11-06 | 2017-12-03 | 2018-11-05 | 2018-12-02 | 2019-11-04 | 2019-12-01 | 2020-11-02 | 2020-11-29 | 2021-11-08 | 2021-12-05 |
| **13** | 2017-12-04 | 2017-12-31 | 2018-12-03 | 2018-12-30 | 2019-12-02 | 2019-12-29 | 2020-11-30 | 2021-01-03 | 2021-12-06 | 2022-01-02 |
