# Supplementary material for: Impact of COVID-19 pandemic on breast and cervical cancer screening in Denmark: A register-based study
Source: eLife. 2023 Mar 21;12:e81605. doi: 10.7554/eLife.81605 (PMC10030107; doi:10.7554/eLife.81605)
Supplement: Supplementary file 2. [file elife-81605-supp2.docx]

Nykøbing Falster Hospital 24 January 2023

**IMPACT OF COVID-19 PANDEMIC ON BREAST AND CERVICAL**

**CANCER SCREENING IN DENMARK:**

**A register-based study**

Mette Hartmann Nonboe^1*^, George Napolitano^2^, Jeppe Bennekou Schroll^3^, Ilse Vejborg^4^  Marianne Waldström^5^ and Elsebeth Lynge^1^

**Supplementary File 2**. Comparing data from present study with data from the Danish Quality Database for Mammography Screening, 6^th^ screening round **(*Danish Quality Database for Mammography Screening. DKMS Report*;** ***2021***) and data from the Danish Quality Database for Cervical Cancer Screening (***Danish Quality Database for Cervical Cancer Screening. DKLS Report 2021*; *2022*)**

| Region | 6^th^ screening round | *DKMS* | Present study | Difference | Difference (%) | DKLS (2021) | Present study | Difference | Difference (%) |
| --- | --- | --- | --- | --- | --- | --- | --- | --- | --- |
| Denmark |  | 577,753 | 605,575 | 27,822 | 4.82 | 372,508 | 323,598 | 48,910 | -13.13 |
| Capital | 01-07-2018 to 01-10-2020 | 159,751 | 161,650 | 1899 | 1.19 | 130,499 | 116,145 | 14,354 | -11 |
| Central | 01-01-2018 to 31-12-2019 | 133,305 | 135,282 | 1977 | 1.48 | 87,520 | 75,967 | 11,553 | -13.2 |
| North | 01-02-2018 to 17-03-2020 | 63,368 | 63,522 | 154 | 0.24 | 35,468 | 32,288 | 3,180 | -8.97 |
| South | 01-08-2018 to 31-08-2020 | 129,879 | 140,591 | 10,712 | 8.25 | 72,850 | 59,738 | 13,112 | -18 |
| Zealand | 01-08-2018 to 19-02-2021 | 91,450 | 104,531 | 13,081 | 14.3 | 46,171 | 39,460 | 6,711 | -14.54 |

*Source: Own calculations based on numbers provided by the Danish Health Data Authority and data from the Danish Quality Database on Breast Cancer Screening*

*The Breast cancer screening rounds run for very different periods in each region. Therefore, the dates for the sixth round are listed. All cervical cancer screening data are reported on yearly basis, from 1 January to 31 December.*
